# Task Modulation of Resting-State Functional Gradient Stability in Lifelong Premature Ejaculation: An FMRI Study

**DOI:** 10.1523/ENEURO.0066-25.2025

**Published:** 2025-06-03

**Authors:** Jiaming Lu, Qing Hu, Yan Lei, Cong Wang, Qiming Deng, Baibing Yang, Qingqiang Gao, Zhengyang Zhu, Ning Wu, Yutian Dai, Xiaozhi Zhao, Xin Zhang, Bing Zhang

**Affiliations:** ^1^Departments of Radiology, Nanjing Drum Tower Hospital, Affiliated Hospital of Medical School, Nanjing University, Nanjing 210008, China; ^2^Andrology, Nanjing Drum Tower Hospital, Affiliated Hospital of Medical School, Nanjing University, Nanjing 210008, China; ^3^Institute of Medical Imaging and Artificial Intelligence, Nanjing University, Nanjing 210008, China; ^4^ Medical Imaging Center, Department of Radiology, Nanjing Drum Tower Hospital, Affiliated Hospital of Medical School, Nanjing University, Nanjing 210008, China

**Keywords:** connectome architecture stability, dynamic functional gradient, fMRI, premature ejaculation

## Abstract

Lifelong premature ejaculation (LPE) is associated with abnormal brain function, as evidenced by functional MRI (fMRI) studies. This study investigates the stability of brain network architectures in resting-state conditions following perturbation by erotic tasks in individuals with LPE. We assessed the resting-state fMRI in the task-free and task-modulated dataset in the 28 right-hand LPE and 17 age-matched normal controls (NCs). The dynamic functional connectome based on the phase-locking algorithm and ROI-wise gradient mapping was compared. The stability of dynamic functional gradient mapping was measure by linear mixed effects across the two datasets in the LPE and NCs. In both groups, the brain functional gradient exhibited a clear transition from unimodal to transmodal in the principal gradient. Additionally, there was a segregation of primary networks observed in the secondary gradient, either before or after the task. In LPE patients, we observed increased stability in the bilateral dorsal prefrontal cortex (*p* < 0.05, Bonferroni corrected) and right temporo-occipital-parietal cortex (*p* < 0.05, Bonferroni corrected) between the pre- and post-task fMRI datasets. The changes of the gradient stability were significantly correlated with the sexual behavior. Our findings suggest that dysfunction in the salience and default mode networks may contribute to delayed recovery from erotic task stimulation in LPE patients.

## Graphical abstract

**Figure:**
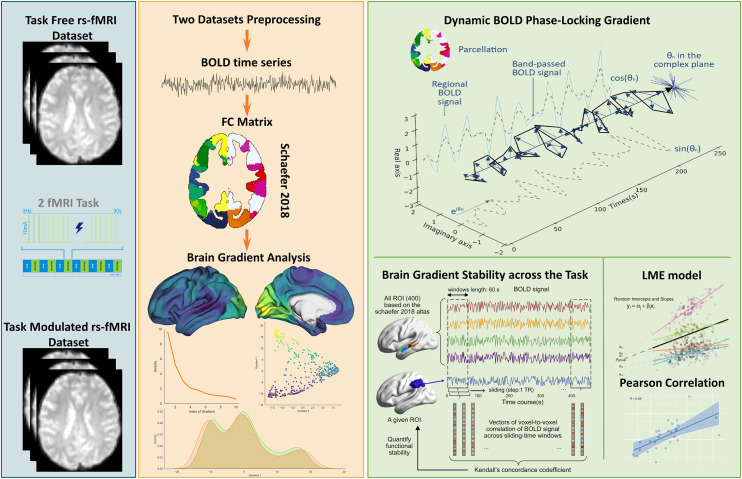
Visual Abstract

## Significance Statement

This study provides novel insights into the neural mechanisms underlying lifelong premature ejaculation (LPE) by examining the task-induced modulation of resting-state functional gradient stability using fMRI. Our findings highlight the critical role of the salience and default mode networks in the impaired recovery of LPE patients following erotic stimulation. By leveraging dynamic functional gradient mapping, we demonstrate that LPE patients exhibit increased stability in the bilateral dorsal prefrontal cortex and right temporo-occipital-parietal cortex, suggesting difficulties in returning to baseline neural states. These results contribute to a deeper understanding of the neural dynamics of sexual dysfunction and may pave the way for targeted therapeutic interventions aimed at improving cognitive flexibility and recovery in affected individuals.

## Introduction

Premature ejaculation (PE) is a prevalent male sexual dysfunction experienced worldwide. As per the guidelines established by the International Society for Sexual Medicine and the European Association of Urology, lifelong PE (LPE) is defined as the consistent occurrence of ejaculation prior to or within ∼1 min of vaginal penetration (Althof et al., 2014). The causes of LPE are multifaceted, encompassing genetics, neurobiology, endocrinology, urology, psychology, and other related factors. In recent times, there has been a growing interest in understanding the neural mechanisms underlying LPE.

Investigating spontaneous brain activity and functional connectivity (FC) using functional MRI (fMRI) provides valuable biomarkers for understanding the neural mechanisms underlying PE (Chen et al., 2021). Studies have observed a reduction in short-range FC density (FCD) in the left orbitofrontal cortex, nucleus accumbens (NAcc), and bilateral middle temporal gyrus among PE patients (Lu et al., 2018). In the region of interest (ROI) level, LPE patients had reduced FC between the NAcc and thalamus, superior temporal cortex, superior temporal pole, inferior frontal gyrus, orbitofrontal cortex, putamen and caudate (Geng et al., 2022). A surface-based regional homogeneity (ReHo) study revealed that LPE patients displayed enhanced ReHo in the right middle frontal gyrus and decreased ReHo in the left triangular inferior frontal gyrus (Xing et al., 2023). These observations shed light on the neural connectivity alterations associated with PE.

However, existing functional neuroimaging studies are mainly concentrated on the brain alteration in one state, whether in terms of changes in brain spontaneous activities (Lu et al., 2018) or erotic picture stimulation task activations (Zhang et al., 2017). There is a notable absence of investigations into the recovery processes of the brain following an erotic stimulation in LPE patients. How quick can the LPE patients’ intrinsic rest brain functional network recovery to the baseline after sexual-related tasks remained unclear.

Functional connectome gradients provide a means to represent connectomes in a low-dimensional space. These gradients are linked to brain network hierarchies and are derived using nonlinear variance decomposition techniques, such as diffusion map embedding (Margulies et al., 2016). The innovative mapping technique, known as connectome mapping, furnished us with a set of gradients illustrating the continuous spatial arrangement of macroscale networks (Margulies et al., 2016; Haak et al., 2018). Studies have shown the existence of a principal connectivity gradient in the macroscale organization of functional brain networks in adults. This primary connectivity gradient delineates the distances among connectivity patterns of brain regions, extending from the primary sensory network to the transmodal regions of the default mode network (DMN; Margulies et al., 2016; Bethlehem et al., 2020). Furthermore, this connectome gradient captures a functional spectrum ranging from perception and action to progressively abstract cognitive domains. This implies that it plays a pivotal role in supporting complex cognitive functions and behaviors (Huntenburg et al., 2018). PE is not only a peripheral physiological condition but also strongly influenced by central nervous system function, psychological factors (e.g., anxiety, attention), and neurochemical imbalances (e.g., serotonin). Connectome gradients capture the system-level integration of these components.

In this study, we aimed to comparing pre- versus post-task resting-state differences in PE patients. The aim of the present study is twofold: (1) we sought to explore the brain network hierarchies by brain gradient in LPE and NC group across pre- and post-task resting-state; and (2) we then explored how brain gradient stability was modified by erotic stimulation. Functional gradient stability for a brain ROI was defined as the concordance of its ROI-level dynamic functional gradient (DFG) over time within a scanning session.

## Materials and Methods

### Participants

This study recruited 28 right-handed LPE patients and 17 age-matched right-handed normal controls (NCs) between 2012 and 2018 at Nanjing Drum Tower Hospital. Further details about the subjects can be found in our previous publications (Zhang et al., 2017; Lu et al., 2018, 2021). The inclusion and exclusion criteria were consistent with those described in previous studies. Lifelong PE patients were diagnosed according to the ISSM guidelines (Serefoglu et al., 2014) and NCs were enrolled with self-reported intravaginal ejaculatory latency time (IELT) of >3 min. All participants exhibited normal erectile function with International Index of Erectile Function (IIEF-5) score >21. The IIEF-5 (Rhoden et al., 2002) and the Chinese Index of Sexual Function for Premature Ejaculation (CIPE)-5 (Yuan et al., 2004) were conducted. This study was approved by the institutional review boards of Nanjing Drum Tower Hospital. Written informed consent was obtained from each participant.

### Data acquisition

Imaging was acquired on a 3T Achieva TX MRI system with an 8-channel head coil. Every subject in this study first went through a resting-state fMRI scan (rs-fMRI) before fMRI task, called task-free rs-fMRI dataset or dataset before task. Then two fMRI tasks were performance. Followed by that, another rs-fMRI scan was acquired, called task-modulated rs-fMRI dataset or dataset after task. Due to this study only focused on the brain change between the two rs-fMRI datasets, for the task description, please see our previous study (Xia et al., 2014; Zhang et al., 2017). The interval between the two rs-fMRI dataset was ∼30 min and the two rs-fMRI scan parameters were as following: the section thickness was 4 mm without any gap between sections. The field of view (FOV) was 192 × 192 mm^2^. The matrix size was 64 × 64. The echo time (TE) was 30 ms. The repetition time (TR) was 2,000 ms. In total, 230 volumes were acquired and each volume comprised 35 transverse slices, covering the entire brain. During the resting-state fMRI scans, participants were instructed to lie quietly with their eyes closed. Additionally, high-resolution 3D T_1_W structural brain images were obtained for each participant. The acquisition parameters for the structural images were as follows: TE, 3,400 ms; flip angle, 8°; TR, 7,600 ms; FOV, 256 × 256 × 192 mm^3^; and slice thickness, 1 mm.

### Data processing

The fMRI data were processed with the MATLAB toolbox Data Processing & Analysis of Brain Imaging (DPABI; http://rfmri.org/DPABI; Yan et al., 2016), which is based on Statistical Parametric Mapping (http://www.fil.ion.ucl.ac.uk/spm). The whole preprocess followed the previous study (Meng et al., 2021). Briefly, we employed a rigid registration to align fMRI volumes with T1-weighted images. Additionally, we performed regression analysis to remove potential confounding effects by regressing out the head motion parameters, cerebrospinal fluid, white matter, and their temporal derivatives (a total of 18 regressors; Power et al., 2012). Participants with a mean fame-wise displacement (FD) exceeding 0.2 mm were excluded from all subsequent analyses. The spatial normalization was performed to the standard Montreal Neurological Institute (MNI) EPI template with a resolution of 3 × 3 × 3 mm^3^. The functional image data were then bandpass filtered within the frequency range of 0.01–0.1 Hz.

### Functional connectome and ROI-wise gradient mapping

Individual ROI-wise functional connectome matrices, based on the Schaefer 2018 atlas (Schaefer et al., 2018), were generated by calculating Pearson's correlation of ROIs time series. This process yielded a FC matrix (400 × 400) for each participant. The BrainSpace Toolbox (https://github.com/MICA-MNI/BrainSpace; Vos de Wael et al., 2020) was used to calculate the gradient mapping. We calculated cosine similarity using the thresholded functional connectome as a similarity measurement. Subsequently, the cosine similarity matrix was input into the diffusion map embedding algorithm, which is a nonlinear dimensionality reduction technique designed to identify gradient components explaining the majority of connectome variances (Coifman et al., 2005). All the settings were maintained at their default values, with *α* set at 0.5 and *t* at 0. Here, *α* regulates the impact of the density of sampling points on the underlying manifold, and *t* controls the scale of eigenvalues of the diffusion operator (Hong et al., 2019).

### BOLD phase-locking dynamic functional gradient

This is a data-driven approach that can obtain the BOLD phase-locking (PL) DFG at each single TR (Alonso Martínez et al., 2020). Initially, the atlas-based dynamic phase-locking (dPL) matrix at each time point was calculated. The dPL estimates the phase alignment between each pair of brain regions, with values ranging from 1 to −1, indicating signals changing in the same or opposite direction, respectively. The gradient mappings were calculated for each single TR for each subject. We utilized Procrustes analysis (Zhang and Zang, 2023), embedded in the BrainSpace Toolbox, to align each individual TR gradient with the group template.

### Computation of stability of dynamic functional gradient mapping

The stability of DFG was calculated based on previous publication (Li et al., 2020). The within-state stability of the DFG for a ROI in the brain was determined by assessing the concordance of the DFG over time within that ROI with the entire brain. The calculation of DFG involved using a sliding-window approach over consecutive data segments, with a window length of 60 s and a sliding step of 2 s, resulting in 201 time windows. The DFG was computed between a specific ROI and all other ROIs within the mask. A higher KCC value for a region indicates that its dynamic functional architecture configuration is more consistent and stable over time.

### Statistical analysis

Statistical analyses were conducted at two spatial resolution levels: the macroscale functional network level and the ROI level. Network-level connectome gradient stability was defined by using a canonical functional network partition scheme (Yeo et al., 2011). Between group difference was examined between patients with LPE and NC in each network across the two time points using linear mixed-effects (LME) model. We also introduced the Bonferroni’s correction to set the significant level (*p* < 0.05/400 = 0.000125). To investigate the clinical relevance of altered connectome gradient stabilities in patients, we correlated the clinical variable (IELT) with the gradient stability in abnormal brain regions. All ROI gradient stabilities correlated to clinical variable using Pearson's correlation.

## Results

### Clinical and demographic characteristic comparisons

There were no differences in age, BMI, marital status, or education level between the groups (Table 1). IIEF-5 scores showed no significant difference between the groups, indicating preserved erectile function in LPE patients. However, LPE patients had significantly lower CIPE-5 scores (*p* < 0.01) and shorter IELTs (*p* < 0.01) than NCs. Furthermore, scores for each CIPE-5 question were significantly lower in LPE patients compared with NCs.

**Table 1.** Demographic and clinical characteristics of patients with PE and control subjects

	LPE patients (*n* = 28)	NC (*n* = 17)	*p*
Age (year)			
Mean ± SD	27.43 ± 4.16	27.59 ± 3.72	0.90
Marital status, *N* (%)			0.77^#^
Single	13 (46.43%)	7 (41.17%)	
Married	15 (53.57%)	10 (58.83%)
Education level, *N* (%)			0.83^#^
Elementary	4 (14.29%)	2 (11.76%)	
High school	9 (31.14%)	7 (41.17%)	
University	15 (53.57%)	8 (47.07%)	
IIEF-5 score			
Mean ± SD	23.86 ± 1.15	24.12 ± 0.70	0.40
CIPE-5 score			
Mean ± SD	8.85 ± 2.10	22.06 ± 2.01	<0.001
IELT (in min)			
Mean ± SD	0.83 ± 0.41	10.75 ± 6.42	<0.001

Data from questionnaires were reported as mean scores (Mean) and standard deviations (SD) for both the LPE and NCs groups. Statistical analysis included chi-squared tests were by the symbol (#), while two-sample *t* tests were employed in its absence. IIEF-5, international index of erectile function; CIPE-5, Chinese index of sexual function for premature ejaculation; IELT, intravaginal ejaculatory latency time.

### Macroscale gradients in patients with LPE and NCs

**Figure 1.**
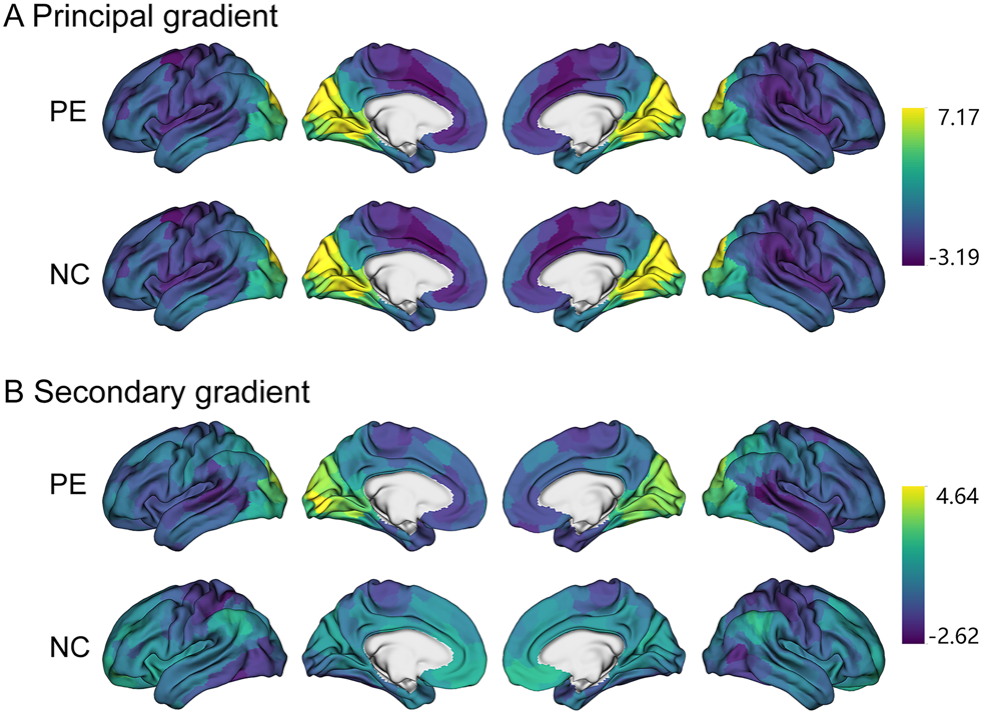
Group-average cortical connectome gradient patterns depicting the principal gradient (***A***) and secondary gradient (***B***), averaged across individuals with LPE and NCs, respectively.

**Figure 2.**
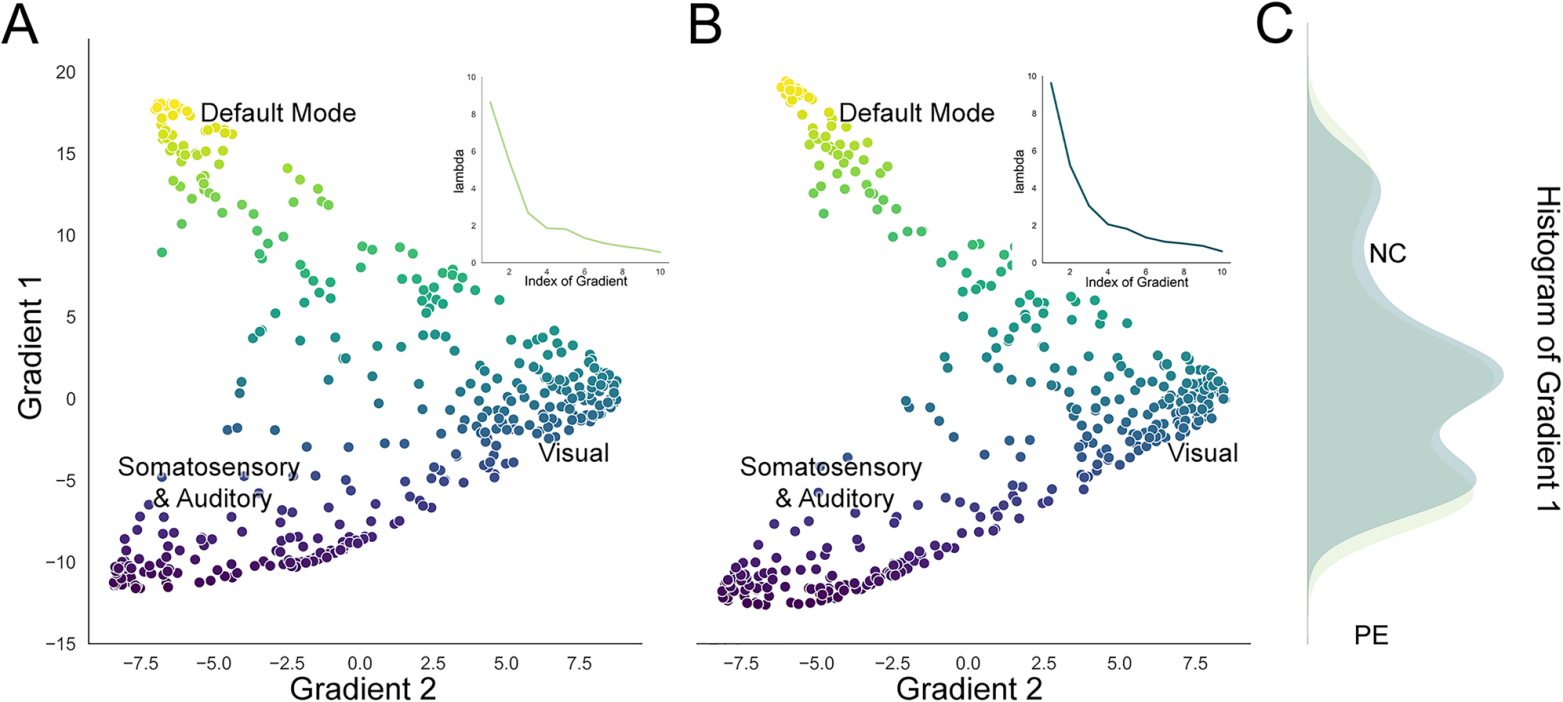
Scatterplot of the first two cortical connectome gradients in the task-free dataset. ***A*** and ***B*** represent NC and lifelong PE, respectively. The *y*-axis represents the principal gradient, exhibiting variation across the default mode network (DMN) denoted by yellow dots to the somatomotor and auditory networks represented by purple dots. The *x*-axis represents the secondary gradient, distinguishing the visual network (teal dots) from the somatomotor and auditory networks (purple dots). The functional networks were defined based on the Schaefer 2018 atlas. ***C***, The distribution of the principal gradient of NC (light green) and PE (teal).

Gradients were derived from individual cortical functional connectomes using the diffusion map embedding method. The first (33.16 ± 5.67%) and the second (22.18 ± 3.17%) functional gradients explained the most variance of the connectomes in the task-free dataset (Fig. 1*A,B*). The first (35.29 ± 5.35%) and the second (20.66 ± 3.91%) functional gradients explained the most variance of the connectomes in the task-modulated dataset. After the alignment procedure, similar to the canonical distribution (Margulies et al., 2016), both LPE patients and NCs in two rs-fMRI datasets demonstrated a distinct transition from a unimodal system to a transmodal system in the principal gradient. Moreover, it revealed the separation of primary networks in the secondary gradient, positioning the visual network on one end and the somatomotor network on the other end (Fig. 2*A*,*B*). There was no significant difference of the explained variance between groups (LPE and NCs) in the principal and secondary both before and after fMRI task (Mann–Whitney *U* test, *p* > 0.05; Fig. 2*C*).

### Network-level gradient stability modified by task

**Figure 3.**
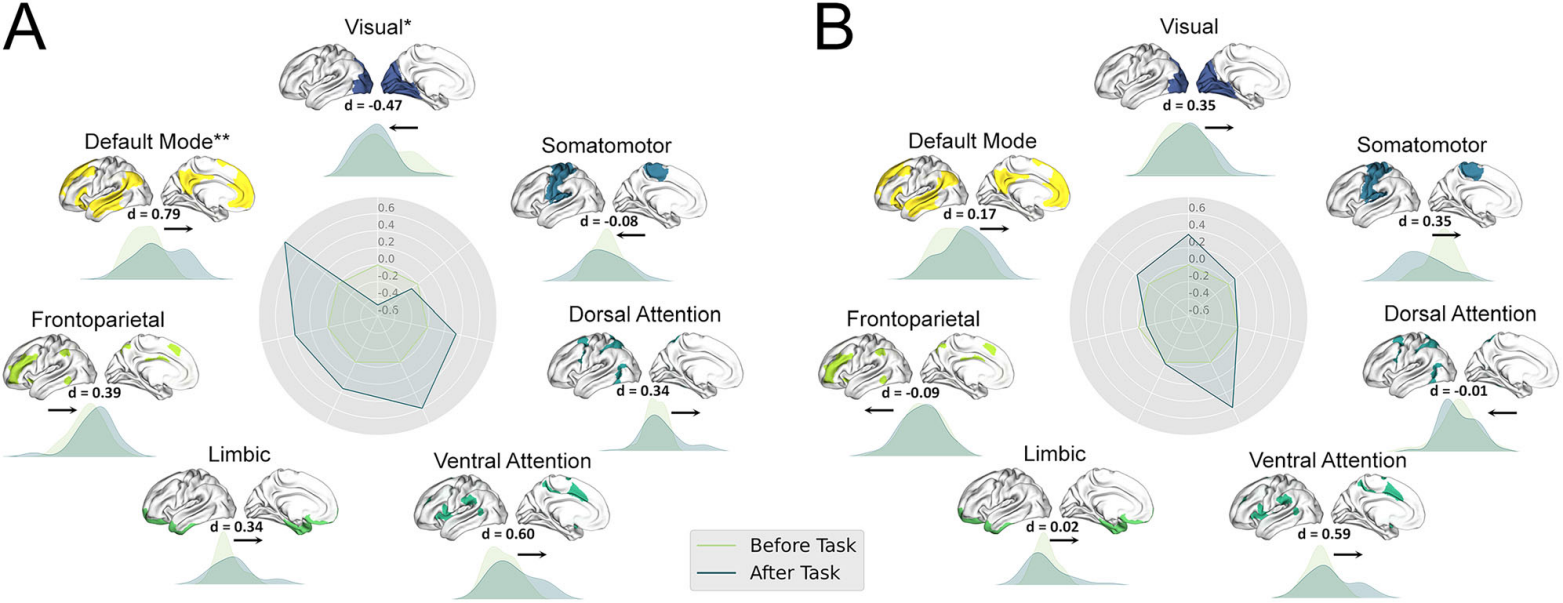
Network-level principal gradient comparisons. ***A***, The radar chart shows the gradient *Z*-score between the task-free and task-modulated rs-fMRI dataset in the LPE group. The visual network declined (*p* < 0.05, Bonferroni corrected), while the DMN significantly increased (*p* < 0.05, uncorrected). ***B***, The radar chart shows the gradient *Z*-score between the task-free and task-modulated rs-fMRI dataset in the LPE group. No significant difference was observed in the NCs group between the task-free and task-modulated dataset. Each network's spatial location was anchored in the corresponding position, and Cohen's *d* was computed.

We used a canonical functional network partition to summarize differences in principal gradient scores between two datasets in LPE patients at the network level. Compared with task-free resting state, there was an apparent increased stability in the DMN of patients with LPE (Cohen's *d* = 0.79, K-S stat = 0.36, *p* = 1.03 × 10^−5^, Bonferroni corrected) and decreased stability in the visual network (Cohen’s *d* = −0.47, K-S stat = 0.25, *p* = 0.031, uncorrected) in the principal gradient after task modulation (Fig. 3*A*). There was no significant difference between the task-free and task-modulated datasets in NC group (Fig. 3*B*). There were no differences between pre- and post-task dataset for gradient scores within the remaining functional networks in the principal gradient in LPE and NC groups.

### ROI-level gradient stability modified by task

**Figure 4. eN-MNT-0066-25F4:**
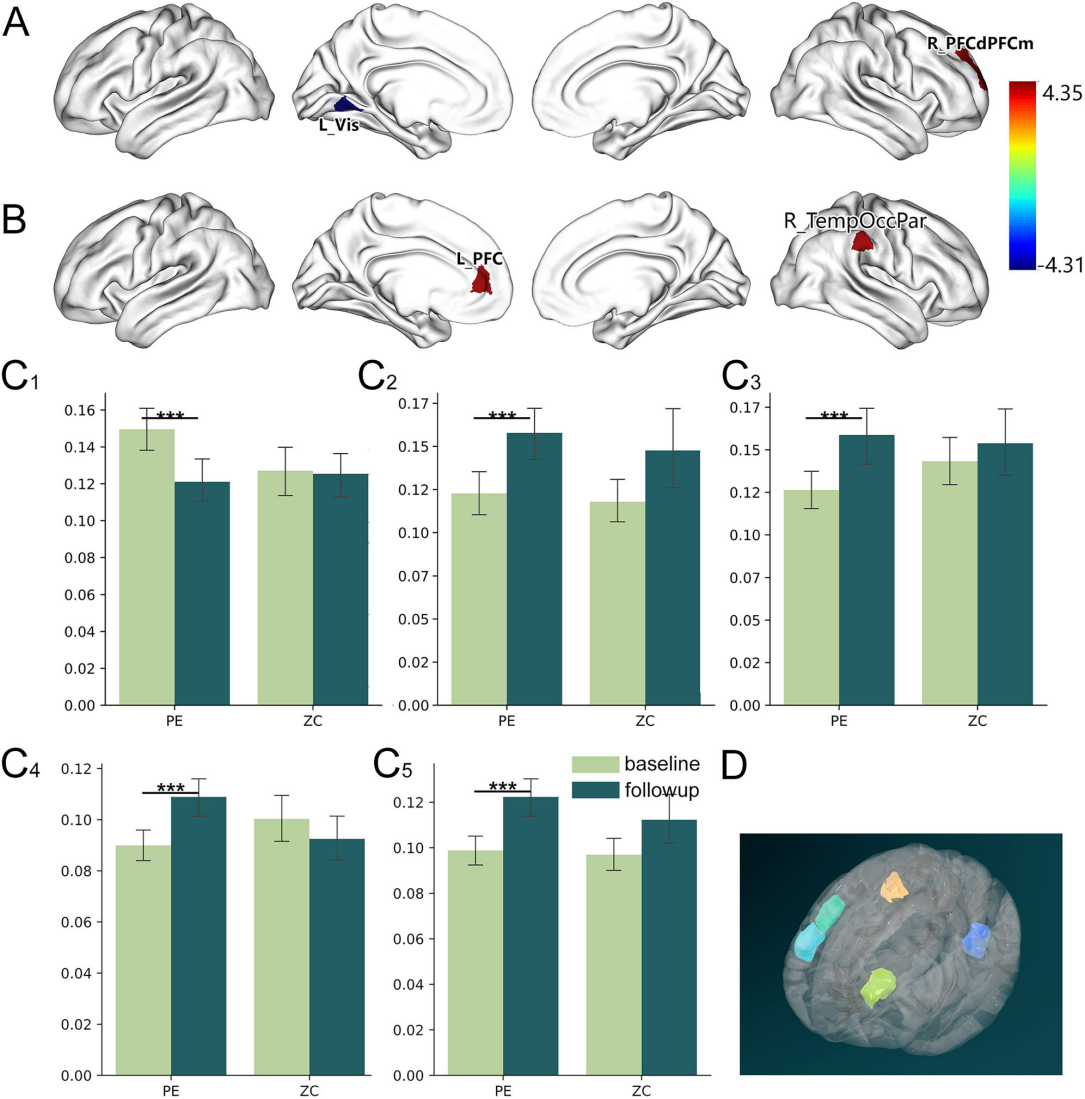
The difference in gradient stability between task-free rs-fMRI and task-modulated rs-fMRI in PE and NC. ***A***, The increased stability in the right dorsal prefrontal cortex and medial prefrontal cortex (PFCdPFCm_5 and PFCdPFCm_8, *p* < 0.05, Bonferroni corrected) and decreased stability in the left visual cortex (Vis_10, *p* < 0.05, Bonferroni corrected) in the principal functional gradient in PE patients between the before and after fMRI task datasets. ***B***, The increased stability in the left prefrontal cortex (PFC_9, *p* < 0.05, Bonferroni corrected) and right temporo-occipital-parietal cortex (TempOccPar_6, *p* < 0.05, Bonferroni corrected) in the second functional gradient in LPE patients between the before and after fMRI task datasets. ***C1–5***, Bar plot of the gradient stability in the five regions of interest (ROIs): left visual cortex (***C1***), right PFCdPFCm_5 (***C2***), right PFCdPFCm_8 (***C3***), left PFC_9 (***C4***), and right TempOccPar_6 (***C5***). ***D***, The 3D render of the glass brain and the five ROIs.

We further compared the principal and second gradient between groups at the ROI level across the two datasets using LME model. Similar to the network-level analysis, the LPE patient and NC showed no significant gradient stability difference between two groups either before or after the task at ROI level (Table 2). There were increased stability in right dorsal prefrontal cortex and medial prefrontal cortex (PFCdPFCm_5 and PFCdPFCm_8, *p* < 0.05, Bonferroni corrected) and decreased stability in left visual cortex (Vis_10, *p* < 0.05, Bonferroni corrected) in the principal functional gradient in PLE patients between the before and after fMRI task datasets (Fig. 4*A,C1–3*). In second functional gradient, there were increased stability in left prefrontal cortex (PFC_9, *p* < 0.05, Bonferroni corrected) and right temporo-occipital-parietal cortex (TempOccPar_6, *p* < 0.05, Bonferroni corrected) in LPE patients between the before and after fMRI task datasets (Fig. 4*B,C4–5*). There were no significant differences across the two datasets in NC of gradient scores within the remaining functional networks in the principal and second gradients.[Fig eN-MNT-0066-25F4][Fig eN-MNT-0066-25F5]

**Table 2.** Differences in dynamic functional gradient stability between before and after task datasets in LPE patients

	ROI index	ROI name	Fixed effects			Main effects		Post hoc test	
			*β*	*t*	*p*	*F*	*p*	*t*	*p*
First gradient	10	L_Vis_10	−0.028	−4.309	9.36 × 10^−5^	7.8483	0.007595	−4.309	0.0001
	383	R _PFCdPFCm_5	0.035	4.353	8.15 × 10^−5^	24.4424	1.216 × 10^−5^	4.353	0.0001
	386	R _PFCdPFCm_8	0.032	4.238	0.000117	11.8843	0.001278	4.238	0.0001
Second gradient	174	L_PFC_9	0.019	4.291	9.9 × 10^−5^	13.9280	0.0005526	4.291	0.0001
	299	R_TempOccPar_6	0.023	4.269	5.04 × 10^−5^	18.807	3.916 × 10^−5^	4.269	0.0001

A linear mixed-effects (LME) model was used in this analysis. Fixed effects, main effects, and post hoc tests were conducted to assess dataset effects. Bonferroni’s correction was applied, setting the significance threshold at *p* < 0.000125 (0.05/400).

### Correlation between clinical scores and gradient stability

**Figure 5. eN-MNT-0066-25F5:**
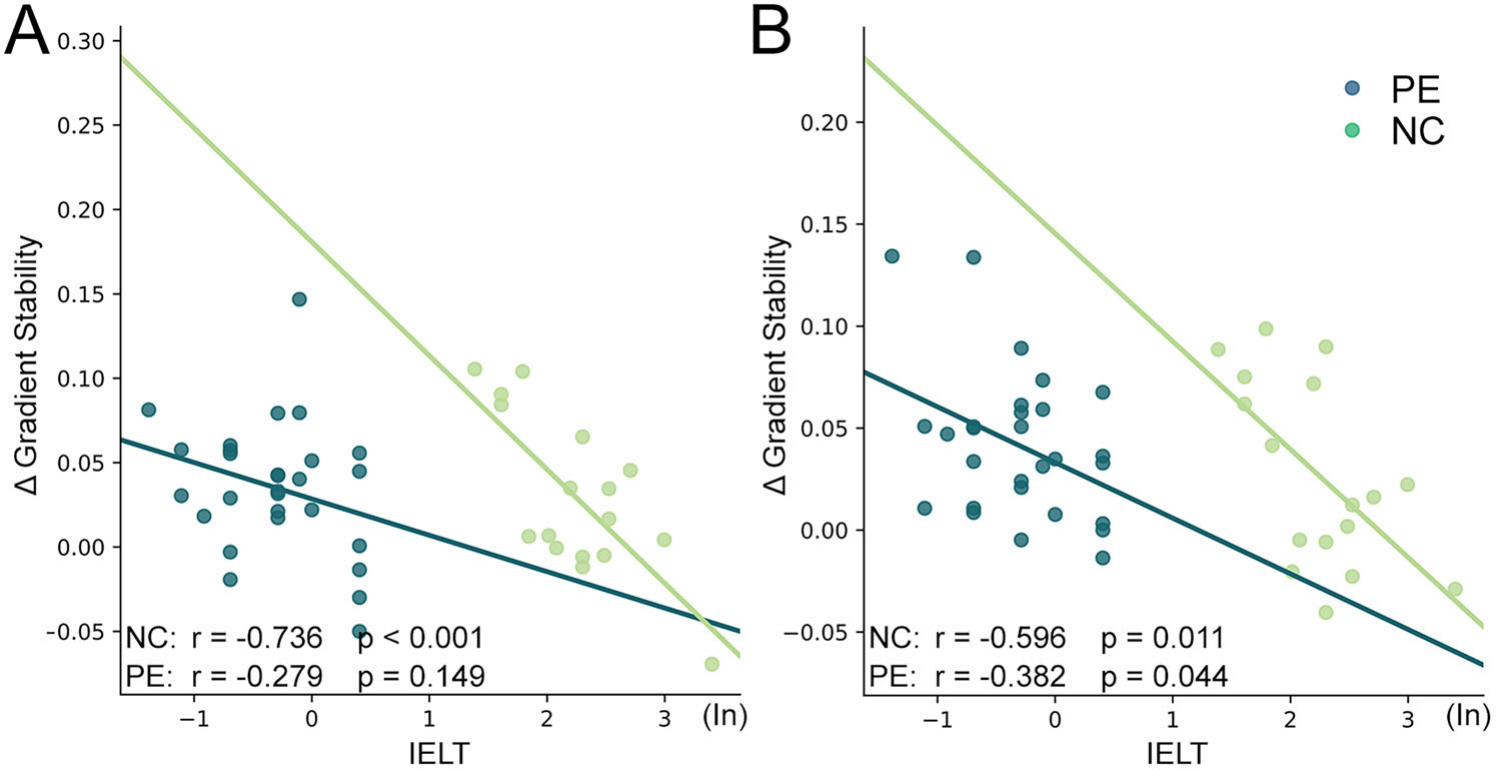
Correlation between clinical scores and gradient stability. ***A***, The correlation between the delta gradient stability in the right PFCdPFCm_5 cortex in the principal functional gradient. ***B***, The correlation between the delta gradient stability in the right TempOccPar_6 cortex in the second functional gradient.

The IELT score was significantly negatively correlated with the delta gradient stability in right PFCdPFCm_5 cortex (*r* = −0.74, *p* < 0.001) in the principal functional gradient in NC group. Also, in the second functional, the negative correlations were found in right TempOccPar_6 cortex both in LPE (*r* = −0.382, *p* = 0.044) and NC (*r* = −0.596, *p* = 0.011) groups. However, the correlation was lower in the patient group than in the NC group (Fig. 5*A,B*).

## Discussion

In this study, we explored the task modulation effects on resting-state fMRI based on the stability of DFG in lifelong PE patient. We found both PE and NC showed a clear transition from a unimodal system to a transmodal system in the principal gradient and separated primary networks in the secondary gradient either before or after the task. The DFG of visual network was significantly decreased and the DMN was significantly increased in LPE patients after the fMRI task modulation, while no significantly alterations were observed in NC group. ROI-level analysis found the DFG stability was decreased in the left visual cortex and increased in the left PFC and right PFCdPFCm and TempOccPar cortex. Further correlation analysis found the IELT score was significantly negatively correlated with delta DFG stability in NC group in right TempOccPar and PFCdPFCm, while in LPE patients, this correlation either reduced or not significant.

We used the brain DFG to measure the brain disturbance in LPE patients. The functional gradients have revealed a primary-to-transmodal gradient in the brain network, documenting a functional spectrum that spans from perception and action to abstract cognition (Xia et al., 2022). Summarizing high-dimensional biological features into low-dimensional manifold representations is a crucial technique for exploring brain function (Katsumi et al., 2023). The existing technology only can explore the static brain functional gradient map, rather than the dynamic functional change. Inspired by Cabral et al., they introduced the Leading Eigenvector Dynamics Analysis to get FC matrix for each single time point in fMRI (Cabral et al., 2017), which aims to study brain dynamics by analyzing fMRI signals as a sequence of discrete phase-locking states that recur over time across all subjects (Cahart et al., 2022). In our study, we use phase-locking technology to detect the brain alteration between the task-free and task-modulated dataset. Then, we introduced the KCC to evaluate the stability of DFG. Our results showed that there was no significant difference between LPE and NCs in macroscale gradient maps, no matter in which dataset. However, the DFG method is more sensitive to detect the post-task effect in brain in LPE patients.

The brain quickly calms down from a task is helpful for us to be prepared for next situation. It requires us have an untouched ability of task switching (Daws et al., 2022). The capability to switch tasks is believed to necessitate extensive high-level cognitive processing, encompassing tasks such as instantiating abstract representations, preparing task-specific processes, and monitoring response selection and execution (Heinonen et al., 2016). Our study suggests that LPE patients exhibit impaired recovery to baseline functional states following two erotic task stimulations compared with NCs. The brain gradient stabilities in bilateral PFCdPFCm and right TempOccPar areas were significantly higher than that in normal control. Previous studies have found that the bilateral PFCdPFCm belongs to the DMN. This network, hierarchically supraordinate, is intrinsic to the brain and is linked to introspection and self-referential thinking (Daws et al., 2022). While the right TempOccPar belongs to salience network, which has been associated with tasks requiring cognitive flexibility such as learning and task switching and primarily implicates in response inhibition, cognitive control, and attention (Cheng et al., 2015), the salience network and the DMN appear to play important roles in creative thought. Dysfunction in these networks has been documented in depression (Goodman et al., 2021) and other disorders characterized by cognitive inflexibility, such as autism spectrum disorder (Watanabe and Rees, 2017) and obsessive–compulsive disorder (Gu et al., 2008). Previous studies in psychogenic erectile dysfunction (pED) patients demonstrated that the abnormally decreased activity at left dorsal prefrontal cortex in patients with pED and the activity intensity of left dorsal prefrontal cortex was positively correlated with the sexual ability and sexual satisfaction (Yin et al., 2020). Our results revealed that the over activity in the PFCdPFCm may result in LPE patients easier to ejaculation. DMN involves in many functions, especially help us prepare for the next task (Pan et al., 2016). So, the quicker the DMN network calms down after a task disturbance, the better for the brain prepared for next task. Our findings indicate that in patients with LPE, the ability of the DMN to rapidly return to a resting state following task engagement is impaired.

This is a single-center cross-sectional study with a small sample size. It could be a challenge to generalize our results to other centers. Also, we did not validate that whether our results were task dependent. Would our results remain the same when we are using different tasks remain unknown. Our study lacks some intervention to see whether the brain regions we detected could be reversion.

### Conclusion

Both PE and NC showed a clear transition from a unimodal system to a transmodal system in the principal gradient and separated primary networks in the secondary gradient either before or after the task. Our findings suggest that dysfunction in the salience and DMNs may contribute to delayed recovery from erotic task stimulation in LPE patients.

## Data Availability

The BrainSpace toolbox was used for functional gradient analysis, which openly available for the community (http://brainspace.readthedocs.io). Seaborn (https://seaborn.pydata.org) has been used in data visualization. Blender was used for 3D brain rendering (https://www.blender.org/). All other data will be made available on request.

## Synthesis

Reviewing Editor: Christine Portfors, Washington State University Decisions are customarily a result of the Reviewing Editor and the peer reviewers coming together and discussing their recommendations until a consensus is reached. When revisions are invited, a fact-based synthesis statement explaining their decision and outlining what is needed to prepare a revision will be listed below. The following reviewer(s) agreed to reveal their identity: Prasanna Karunanayaka. Note: If this manuscript was transferred from JNeurosci and a decision was made to accept the manuscript without peer review, a brief statement to this effect will instead be what is listed below.

This is an important and interesting study with only a few minor comments to address.

- Please explain the biological significance of connectome gradient and why this metric is better suited for PE studies. This is very briefly discussed in the introduction. The paper seems to be presented in a very abstract manner with limited translational value.

- There are grammatical mistakes that needs to be corrected.

- Is the statistical analysis corrected for multiple comparisons? Please explain your choice and examine if it is the most appropriate.

## Author Response

Responses to the reviewers' comments:

We are sincerely grateful to the editors and reviewers for their thoughtful and constructive comments. These insights have been invaluable in enhancing the quality of our manuscript and providing important direction for future research. We have carefully addressed each comment and revised the manuscript accordingly. Below is a summary of the main changes and detailed responses to each comment.

Reviewers' comments - Please explain the biological significance of connectome gradient and why this metric is better suited for PE studies. This is very briefly discussed in the introduction. The paper seems to be presented in a very abstract manner with limited translational value.

Reply: Thank you for the suggestion. The connectome gradient is a novel and biologically informed framework that describes the organization of the brain's connectivity in terms of smooth, continuous transitions from primary sensory/motor regions to higher-order transmodal areas (like the default mode network or DMN). This reflects the brain's functional hierarchy, where sensory input is progressively integrated into abstract cognitive and emotional processes. PE is not only a peripheral physiological condition but also strongly influenced by central nervous system function, psychological factors (e.g., anxiety, attention), and neurochemical imbalances (e.g., serotonin). Connectome gradients capture the system-level integration of these components. We have added this description to the manuscript to emphasize the biological significance of the connectome gradient.

- There are grammatical mistakes that needs to be corrected.

Reply: Thank you for pointing this. We thoroughly reviewed and revised the manuscript.

- Is the statistical analysis corrected for multiple comparisons? Please explain your choice and examine if it is the most appropriate.

Reply: Thank you for the question. We introduced the Bonferroni correction by setting the significance threshold at P < 0.000125 (i.e., 0.05/400) in this study to account for multiple comparisons. Several multiple comparison correction methods exist, such as FDR and TFCE. Among these, the Bonferroni correction is considered one of the most stringent approaches. We selected this method to enhance the reliability and robustness of our findings.
